# Transfer market activities and sportive performance in European first football leagues: A dynamic network approach

**DOI:** 10.1371/journal.pone.0209362

**Published:** 2018-12-19

**Authors:** David Matesanz, Florian Holzmayer, Benno Torgler, Sascha L. Schmidt, Guillermo J. Ortega

**Affiliations:** 1 Applied Economics Department, University of Oviedo, Oviedo, Spain; 2 WHU–Otto Beisheim School of Management, Center for Sports and Management (CSM), Düsseldorf, Germany; 3 The School of Economics and Finance, Queensland University of Technology, Brisbane, Australia; 4 CREMA–Center for Research in Economics, Management and the Arts, Zürich, Switzerland; 5 Science and Technology Department, Universidad Nacional de Quilmes and CONICET, Bernal, Argentina; Queen Mary University of London, UNITED KINGDOM

## Abstract

Professional football is a globalized game in which players are the most valuable assets for clubs. In this study, we explore the evolution of the football players’ transfer network among 21 European first leagues between the seasons 1996/1997 and 2015/2016. From a topological point of view, we show that this network achieved an upper limit expansion around season 2007/2008, thereafter becoming more connected and dense. Using a machine learning approach based on Self-Organizing Maps and Principal Component Analysis we confirm that European competitions, such as the UEFA Champions League or UEFA Europa League, are indeed a “money game” where the clubs with the highest transfer spending achieve better sportive performance. Some clubs’ transfer market activities also affect domestic performance. We conclude from our findings that the relationship between transfer spending and domestic or international sportive performance might lead to substantial inequality between clubs and leagues, while potentially creating a virtuous (vicious) circle in which these variables reinforce (weaken) each other.

## Introduction

Professional football is regarded as the most popular sport throughout the world, famous for both its players and its clubs. Several football players are recognized as international superstars, including Cristiano Ronaldo, Lionel Messi, Neymar, or Paul Pogba, while less renowned players are also essential to delivering the final team ‘product’ of football on the pitch. Accordingly, players constitute the most valuable asset for football clubs, particularly for top clubs such as Real Madrid, FC Barcelona, Paris Saint-Germain, or Manchester United. Regardless of the reputation of players or clubs, the fundamental aim of both is to achieve outstanding sportive performance, as it is the essence of the clubs’ financial performance and survival.

Sportive performance in football is ultimately achieved by a squad of approximately 24 players and the corresponding team of trainers. Thereby, a football team can be built in two different ways, which are often seen in combination: First, football players can be ‘grown’ within a club. Here, talents are recruited at very young age due to their potential skill set. Subsequently, they receive in-depth football education by going through different youth teams until they are ready to make the move to the professional team. Second, players can be purchased or loaned on the international transfer market. These transfer market activities have increased significantly, reaching a record gross transfer spending of €4.38 billion by European top-league clubs, with 81% of that amount concentrated within the first leagues of England, Germany, Italy, and Spain [1].

At a club level, the global football transfer market has seen stratospheric figures in terms of transfer fees paid for individual players. For instance, the transfer of Neymar from FC Barcelona to Paris Saint-Germain in summer 2017 was for the record amount of €220 million, making him the most expensive player in the history of professional football. This sharp increase in transfer prices has attracted the attention of the media, highlighting the divergence of transfer fees paid and the ‘real’ player value with some even describing this development as ‘hyperinflation’ [2]. The effects of such transfer market activities on sportive performance have become a much-discussed topic in the football industry but also in academia. Currently, richer clubs seem to win more often and, as a result, uncertainty about match outcomes and end of season league ranks has fallen over time. As such, the question remains as to whether a costly transfer market strategy is the best way to achieve sportive performance.

The football transfer market has been studied in academia for decades [3–6]. However, recent availability of data and the increased interest in transfer market economics has accelerated research on this topic. For instance, the football transfer market has been employed to analyze labor mobility and globalization [4, 5, 7–9], football player transfer ‘prices’ [7, 10, 11], or the relationships between the clubs’ transfer market activities and sportive performance [6, 12].

Recent studies have improved our understanding of the transfer market-performance relationship. Liu et al. [12] analyze 24 top football leagues from 2011 to 2015 and conclude that professional football is a money game where larger transfer market investments are positively related to sportive team performance. Thereby, they identify different league categories such as ‘money’, ‘farm’, and ‘outlier’ leagues. ‘Money’ leagues reveal a negative correlation between the clubs’ annual transfer balance and average league game points obtained. Hence, the more the clubs within this league type spend on transfers, the more their match performance increases. In contrast, this correlation is found to be positive in ‘farm’ leagues, since the higher the clubs’ transfer market profits, the better match performance becomes. The remaining leagues are classified as ‘outlier’ leagues. Remarkably, the authors identify a large and increasing inequality within the transfer market. Similarly, [6] analyze the transfer market history from 1990 to 2015. They cluster clubs into certain profiles and find evidence that regardless of the clubs’ budgets, transfer market strategies deeply affect sportive performance. They conclude that clubs need to trade globally in the transfer market if they want to dominate their domestic league.

Based on these findings, we explore the European transfer market between seasons 1996/1997 and 2015/2016. We focus our analyses on the first leagues from 21 countries. Similar to the work conducted by Liu et al. [12] and Rossetti and Caproni [6], we employ several network measures and a complex system approach to study the evolution of the transfer market over the 20-year period. We generate descriptive measures of the network structure that characterize its evolution over time. Additionally, we employ a machine learning approach based on Kohonen Self-Organizing Maps (SOMs) to show relative similarities among clubs in the multi-dimensional space, maintaining the original topological relations [13, 14]. Principal Component Analysis (PCA) is performed to analyze the relative importance of our node variables. Detailed explanations on the methodology are presented in the following section.

The aim of the paper is to analyze the relationship between the clubs’ transfer market activities and sportive performance. Thereby, the contributions of the paper are twofold. First, we apply a network approach and extend previous analyses by introducing a dynamic perspective on the topological characteristics of the European transfer network over 20 years. Second, the machine learning approach based on SOMs and PCA allow us to explore the relationships between clubs’ transfer market activities and sportive performance over time. Our results describe the evolution of the transfer network and show that financial resources used to acquire football players are a decisive variable in explaining sportive performance in some domestic leagues but not in others. Remarkably, only clubs that invest substantial financial resources reach top positions at UEFA level.

The remainder of this article is organized as follows. First, we introduce the dataset and methodology. Second, we present the results of our network analyses. Here, we illustrate the connection between transfer market activities and sportive performance. We conclude with our key findings, discuss the inequality in financial resources among leagues and clubs, and provide future research directions.

## Data and methodology

We studied the football player transfer market activities among European first leagues from 21 countries (EFL21) between seasons 1996/1997 and 2015/2016. These include Austria (AUT; name of examined league: Bundesliga), Belgium (BEL; Jupiter Pro League), Croatia (CRO; 1. HNL), Denmark (DEN; Superligaen), England (ENG; Premier League), France (FRA; Ligue 1), Finland (FIN; Veikkausliiga), Germany (GER; Bundesliga), Greece (GRE; Super League), Hungary (HUN; NB I.), Italy (ITA; Serie A), The Netherlands (NED; Eredivisie), Norway (NOR; Eliteserien), Poland (POL; Ekstraklasa), Portugal (POR; Liga NOS), Russia (RUS; Premier Liga), Scotland (SCO; Premiership), Spain (ESP; La Liga), Switzerland (SUI; Super League), Turkey (TUR; Süper Lig) and Ukraine (UKR; Premier Liga). Of these, the English Premier League, Spanish LaLiga, German Bundesliga, Italian Serie A, and French Ligue 1 are the most prominent leagues, often referred to as the ‘big five’ leagues in Europe. Within this time period, the studied transfer market activities include more than 135,000 transfers to and from football clubs of these 21 leagues and other national or international leagues.

For every transfer, we collected data including the specification of the respective transfer, such as the club before and after the actual transfer, transfer fee or loan fee, as well as player market value, field position, age, and nationality. This dataset was enriched with clubs’ national performance, measured as end of season league ranks, and international performance, measured with the UEFA club coefficients. The latter is based on a club’s points obtained at European club competitions such as the UEFA Champions League and UEFA Europa League. The transfer market data was retrieved from the transfer market website www.transfermarkt.de. National performance data was derived from www.transfermarkt.de, www.kicker.de, www.soccerway.com, www.weltfussball.de or www.weltfussball.com, while international performance data was sourced from www.uefa.com and https://kassiesa.home.xs4all.nl.

With the above information, a transfer network was constructed for every season with clubs as nodes and player transfers among clubs/nodes during the respective season as links. The data included also transfers towards the end of the players’ careers. Hence, a node called ‘end of career’ was included. In addition, missing information regarding source and target clubs was found in the dataset. Thus, an ‘unknown’ node was included. However, transfers from or to these nodes only accounted for less than 0.3% of all transfers. Within this network, links were weighted by the fees paid (or received) for each transfer. The network included ‘core nodes’, which incorporated the EFL21 clubs, and ‘neighboring nodes’, which involved clubs that received or delivered players from or to the ‘core nodes’ respectively. Note that not only players moved throughout every season, but ‘core nodes’ also changed due to promotions or relegations of clubs to or from the respective first league. By studying the yearly constructed networks sequentially over time, a dynamic view of the transfer market was generated. In sum, for each node and their links the following attributes were accumulated:

- Nodes (clubs): Name, country, transfer spending (transfer spending is the amount of money each club has spent in transfers in each season, while transfer earnings represent the amount of money each club has generated from transfers in each season), transfer fee volume (transfer spending + transfer earnings), league, domestic rank, UEFA points, continent
- Links (players transferred): Transfer fee, name, age, nationality, field position

We calculated several of the most commonly used measures of complex network properties. For this, unweighted directed networks for each season were generated. As such, the weights from transfer/loan fees were neglected since the analysis focused purely on the topological properties of the transfer network. Based on the R package igraph, important network measures were calculated, such as the Average Path Length (APL), the Density of Links (Density), and the average Clustering Coefficient (CC) [15]. The APL is defined as the average of the shortest paths between every pair of nodes in the network in terms of the number of steps along the nodes. In this sense, low values of the APL implied a highly connected topology. Density is defined as the ratio of the actual number of links in the network to the number of all possible links among the network nodes. Lastly, the average CC quantifies the degree to which network’s nodes are inclined to cluster together. High values of CC implies the existence of rather dense subgroups of network nodes. Extended explanations on network property measures can be found in S1 Text.

Further, a machine learning approach was employed to deal with the multivariate information attached to every club. For this, the following five variables of every EFL21 club in every season were taken into account:

- Transfer spending
- Transfer balance: Transfer earnings from minus transfer spending on player transfers in the respective season.
- Domestic rank
- UEFA points
- Relative transfer spending: A coefficient of relative transfer spending in relation to the overall transfer spending in the corresponding league during the respective season.

We tracked the evolution of these five variables associated to every club across the entire time period. The best way to map such five-dimensional data visually while maintaining the original closeness relations is through the Kohonen SOMs [13, 14, 16]. This algorithm approximately preserves neighboring relationships among points (i.e. clubs’ characteristics) in the high-dimensional input space when mapped onto a two-dimensional space. In this way, original patterns of the (dis-) similarities among clubs and relationships with the variables considered are visualized in a two-dimensional plot. With Euclidean distance, all variables were normalized. Kohonen self-organizing maps have been extensively applied to clustering problems and data exploration in linguistic, artificial intelligence, natural sciences, and more recently, business and finance contexts [17, 18]. As a final step, PCA [16] was employed to quantify the relative weight of each variable. In addition, the domestic rank variable was rescaled to assign higher numbers to better final positions. A ‘one’ was assigned to the top position and a ‘zero’ to the last position, while linear interpolation was conducted for the positions in-between.

## Results

### Transfer market characteristics

**Fig 1:**
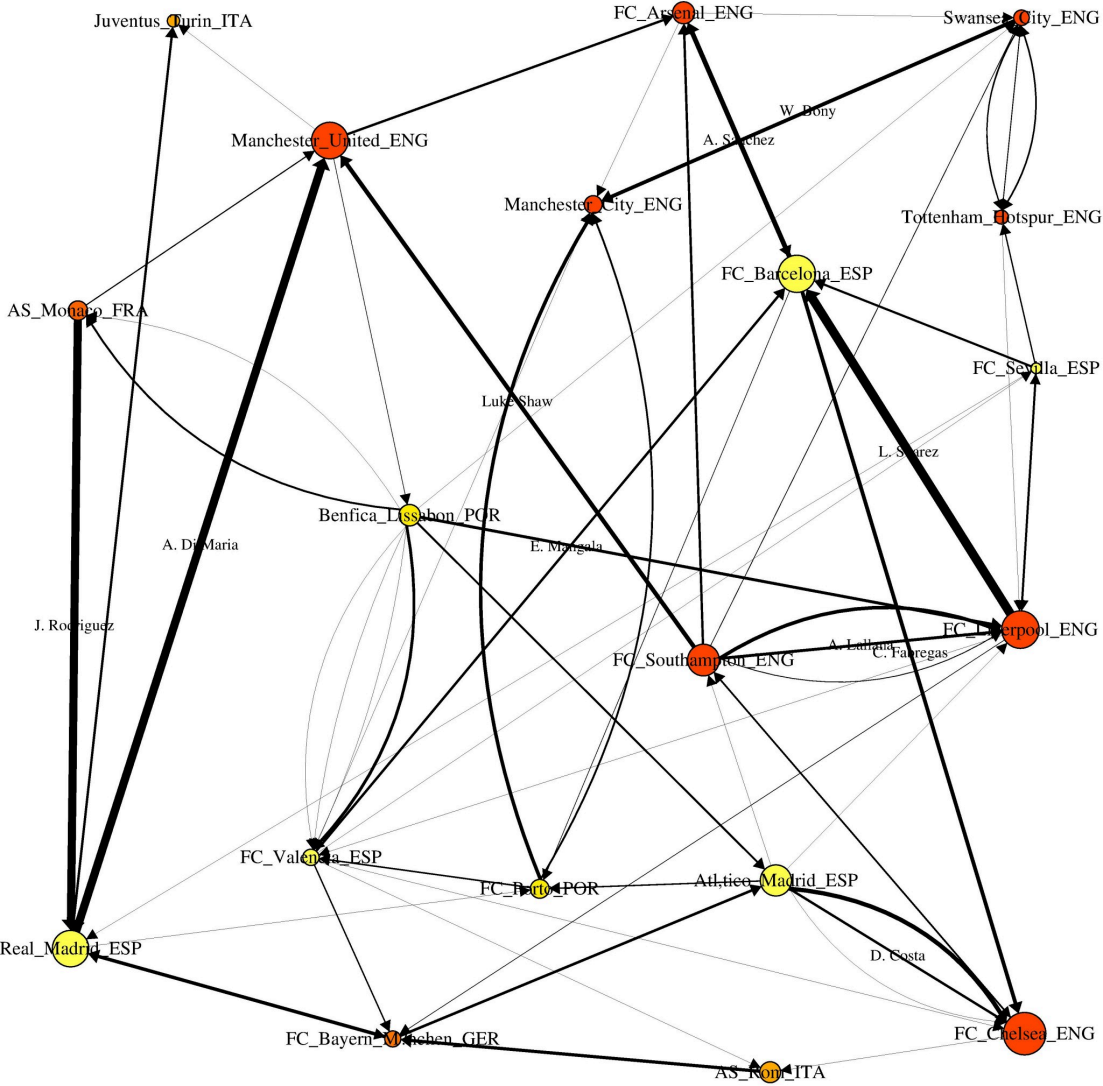
Transfer sub-network for season 2014/2015. Transfer market activities among clubs that have transferred players (in and out) with an overall transfer fee volume greater than €70 million are illustrated. Nodes’ (clubs’) colors correspond to the league, while node sizes correspond to the total transfer fee volume traded by every club. The widths of links are proportional to player costs. Only the names of players transferred for more than €30 million are displayed.

Fig 1 illustrates a typical transfer sub-network for season 2014/2015. This network shows the transfer market activities among clubs that have transferred players (in and out) with an overall transfer fee volume greater than €70 million during this season. Nodes’ (clubs’) colors correspond to the league and node sizes correspond to the total transfer fee volume traded by every club. Links width are proportional to player costs. The names of the most expensive transfers (more than €30 million) are displayed. As expected, English clubs dominate the network followed by Spanish, Italian, and Portuguese clubs. As such, only two clubs from France (AS Monaco) and Germany (Bayern Munich) appear in the network. These two countries are, however, more frequently represented in other seasons. In season 2014/2015, the transfers of Di Maria (transferred from Real Madrid to Manchester United), Luis Suarez (FC Liverpool to FC Barcelona), and James Rodriguez (AS Monaco to Real Madrid) are the most expensive. Of course, the global network for all transfers made in a particular season is much larger with more than 8.000 transfers among 2.200 clubs.

**Fig 2:**
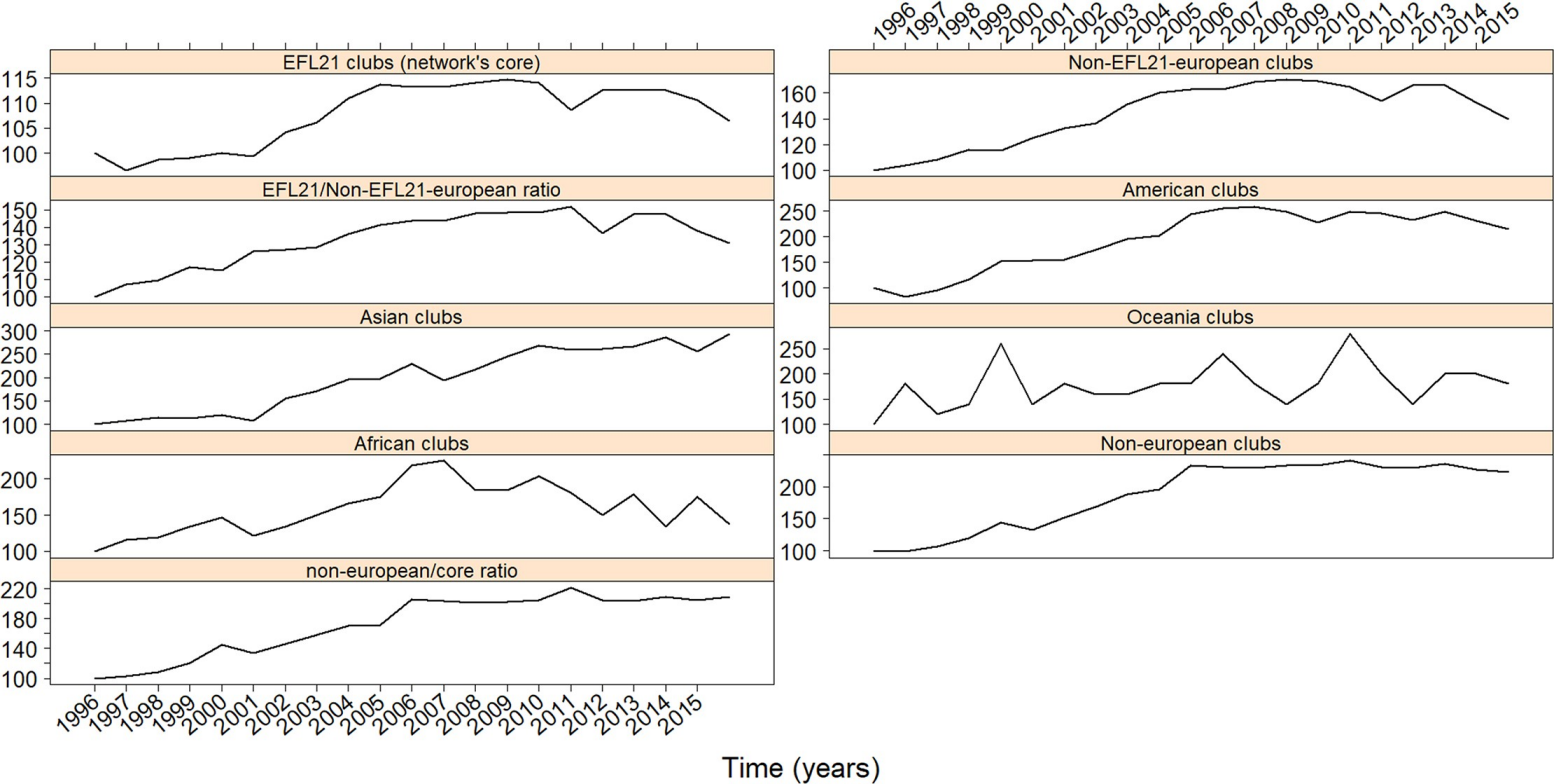
Evolution of transfer market activities with EFL21 clubs (‘core nodes’) for different geographical regions (fixed base). Each panel describes the evolution of the number of regional clubs involved in transfer market activities with EFL21 clubs. Years in the x-labels correspond to the first year of the season (i.e. 1996 for 1996–1997 season).

Fig 2 displays the evolution of the number of participating clubs in the transfer network across different geographical regions. The number of EFL21 clubs in the transfer market, the ‘core nodes’ of the network, has reached a maximum around season 2007/2008 followed by a slight decline thereafter (upper left panel of Fig 2). Throughout most regions, we observe similar patterns. However, transfers from/to Asian clubs also increase after season 2008/2009. In contrast, African clubs experience an extraordinary decline in their transfer market activity with EFL21 clubs. Interestingly, an increasing number of players of African descent have started to return to Africa recently in order to play in their home regions [19]. Overall, taking into account only the number of transfers and clubs involved, the network seems to have reached a tipping point just at the time of the recent financial crisis, while only the interaction with Asian clubs presents an exception. S1 Fig shows the chained index for the same geographical regions, which includes a comparison among successive calculation periods and, therefore, focuses on the network evolution from year-to-year. Similarly as before, this figure shows that most regions observe a downward trend over time, while Asia and Oceania remain as exceptions.

**Fig 3:**
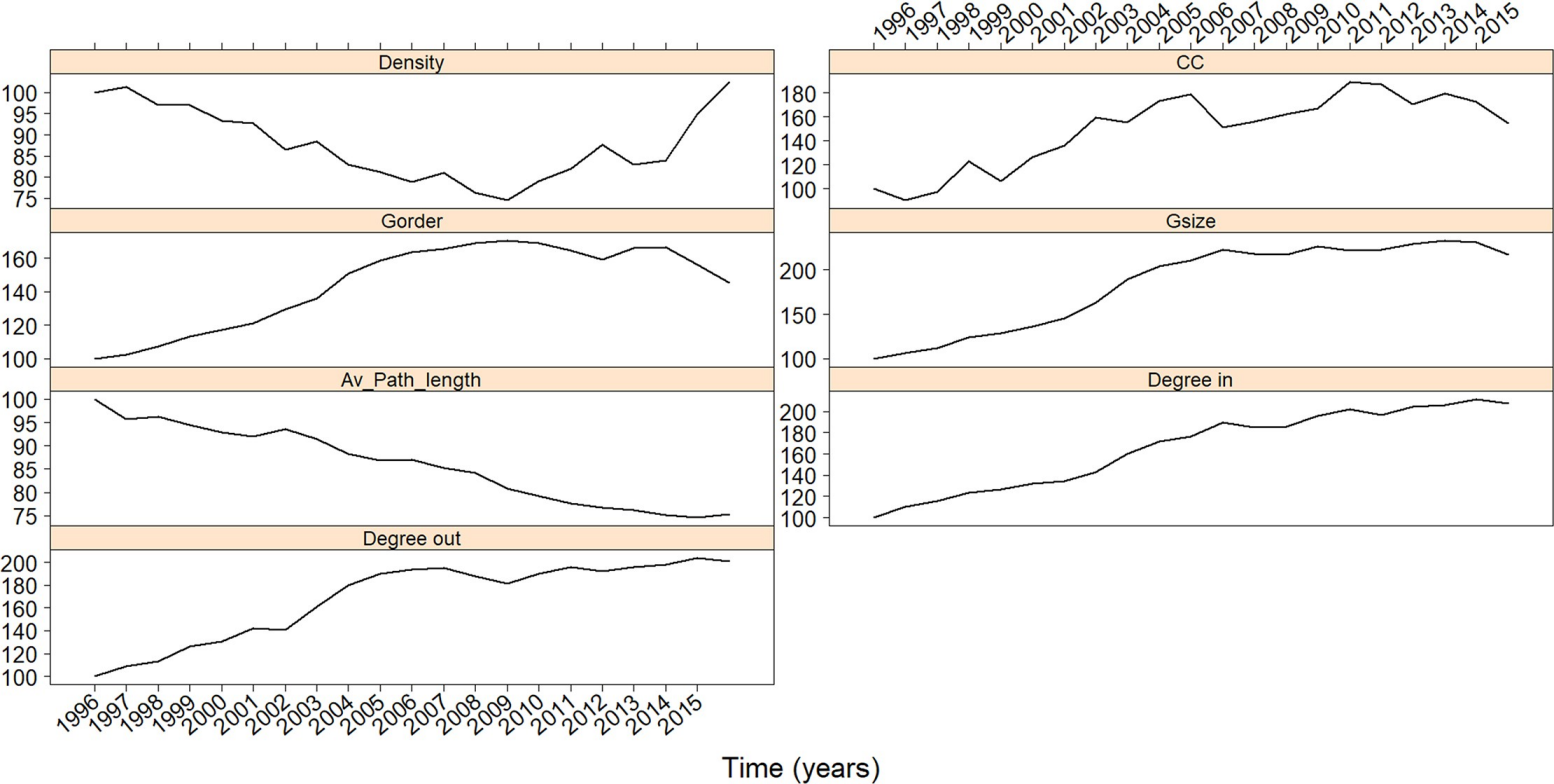
Evolution of the network measures (fixed base). Different descriptive network measures. Density: density of links/transfers, average (over the network nodes/clubs). Clustering Coefficient, Gsize: number of network links/transfers, Gorder: number of network nodes/clubs, Degree-in: average number of incoming links/transfers per club, Degree-out: average number of outgoing links/transfers per club. Years in the x-labels correspond to the first year of the season (i.e. 1996 for 1996–1997 season).

Fig 3 shows the evolution of different network measures. Gorder (the number of network nodes) and Gsize (the number of network links) characterize respectively the number of clubs involved in the network and the actual number of transfers between them. Both measures show a steady increase until season 2007/2008 and stagnation (Gsize) or decline (Gorder) afterwards. However, the ratio of the number of transfers to the number of clubs (Gsize/Gorder) displays a steady increase from the beginning of the analyzed period until 2016. Concordantly, the evolution of Density shows a continuous decline while the number of clubs and transfers involved in the network increase before 2007/2008, and reveal a sharp uplift thereafter. Consequently, after a period of a growth with less connection, the network begins to slowly diminish in size, reaching a higher grade of connectivity. This development is also apparent in the APL which shows a steady and rather strong decrease (20% since 1996), implying an increasing connectedness of the transfer network where nodes get ‘closer’ to each other over time. Note also the increase in average CC, which has almost doubled in value since the beginning of the period analyzed. Hence, the transfer network evolves towards a clusterized network. These findings imply that the transfer network evolves towards a small-world network [20], as anticipated by Liu et al. [12]. Likewise, the average number of incoming (degree-in) and outgoing links (degree-out) per club has slowly grown throughout the time period. Finally, the CC has doubled its initial value at the end of the period. The initial CC value (season 1996) was around 0.1 while the CC reached approximately a value of 0.2 in season 2015, which is in line with results by Liu et al. [12] for the years 2011 to 2015. S2 Fig shows the chained index of the same measures. APL and Density measures experience an upward trend, reinforcing the idea of a potential transformation from a dynamic to a more connected network.

### Transfer market activities and sportive performance

**Fig 4:**
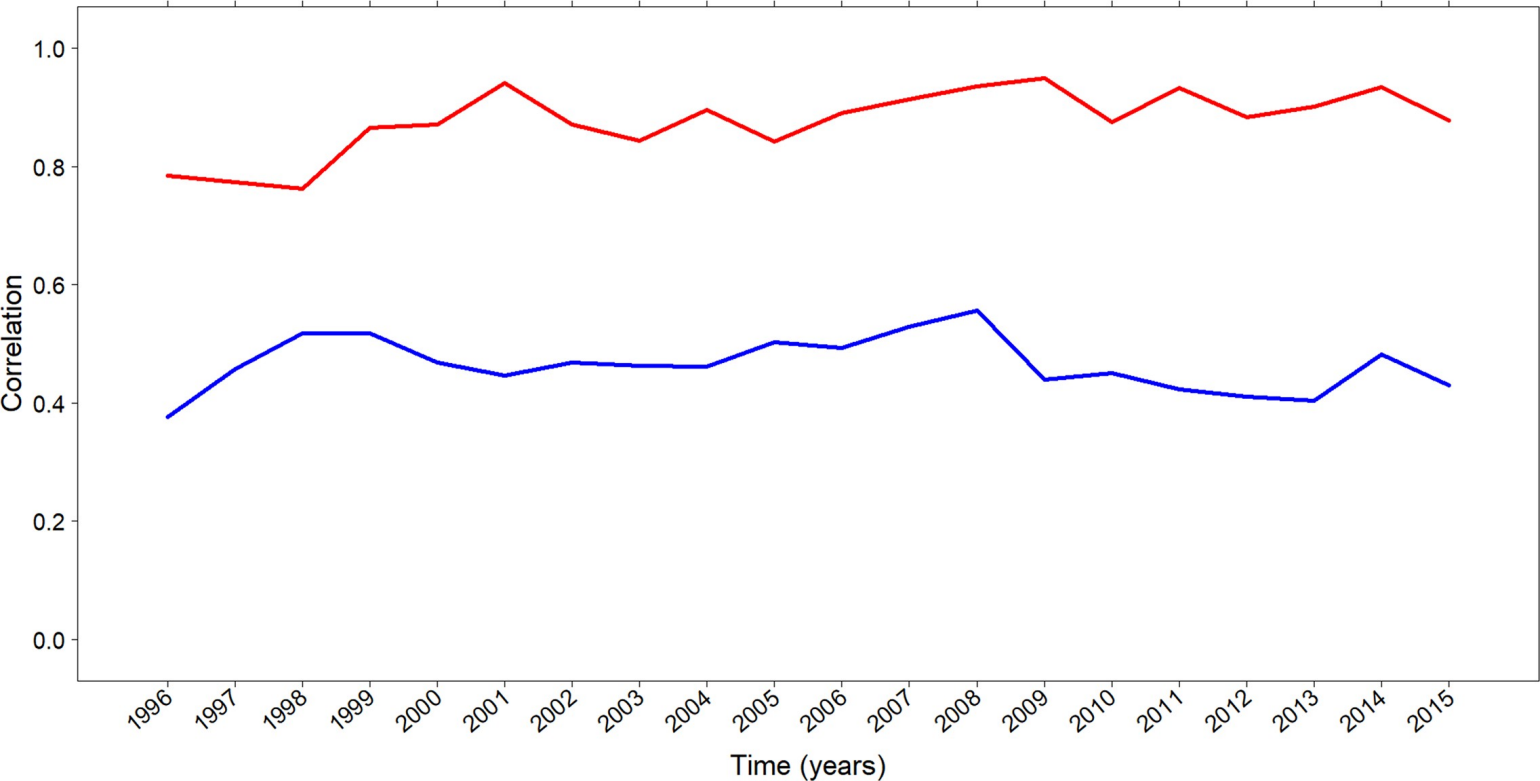
Transfer spending and UEFA points. Correlations between leagues’ total transfer spending and UEFA points (all clubs; blue line) and correlations between clubs’ total transfer spending and UEFA points obtained irrespective of their corresponding league (red line). Years in the x-labels correspond to the first year of the season (i.e. 1996 for 1996–1997 season).

In this Section we study the relation between transfer market activities and international sportive performance at both league and club levels. Fig 4 shows the correlations between transfer spending and international sportive performance, measured by UEFA points. As such, the blue line shows the correlations between the net amount of transfer spending by all clubs of a league and the corresponding total amount of UEFA points that all clubs of a league achieved in the respective season. It is apparent that correlations remain rather flat over time and fluctuate between values of 0.42 and 0.52. Even though the overall level of correlations is high, it does not reveal fully convincing evidence that international performance can be achieved through high investments on the transfer market. The red line in Fig 4 presents the same correlation at club level and shows clubs’ transfer spending and the corresponding earned UEFA points irrespective of their corresponding league. Here correlation coefficients reach values between 0.80 and 0.95. This indicates more clearly that performance in UEFA competitions is money-driven as only clubs with sufficient financial resources seem to be able to achieve substantial sportive performance.

**Fig 5:**
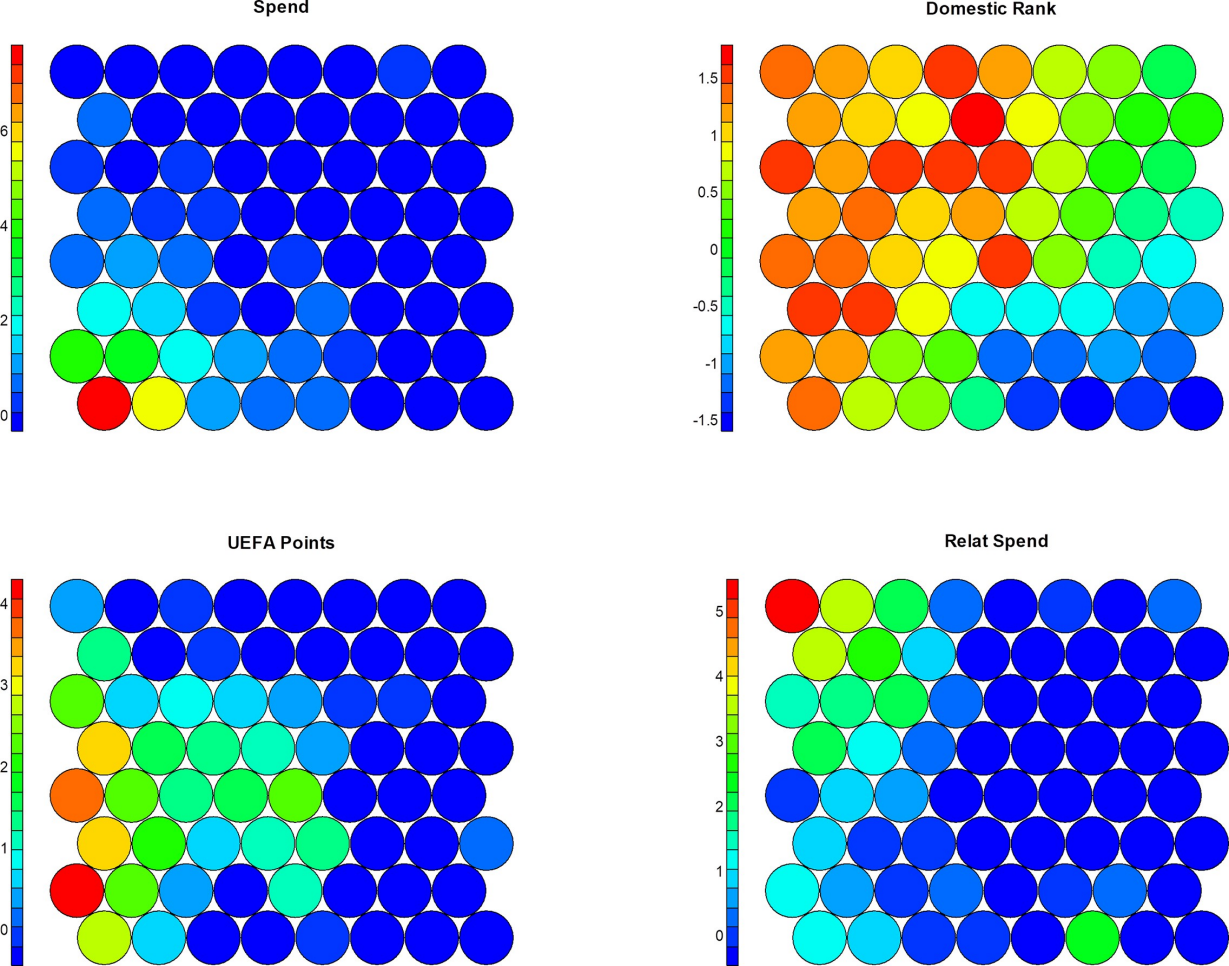
Kohonen SOMs for season 2001/2002. Examined variables: transfer spending (*Spend*), domestic rank (*Domestic Rank*), UEFA points (*UEFA Points*), relative transfer spending (*Relat Spend*) (all scaled).

In addition, a multivariate analysis was performed using the Kohonen SOMs. By considering five characteristics–transfer spending (*Spend*), domestic rank (*Domestic Rank*), UEFA points (*UEFA Points*), relative transfer spending (*Relat Spend*), and transfer balance (*Balance*)–SOM maps were constructed for each season. Fig 5 displays selected Kohonen maps for the first four variables in season 2001/2002. In each panel it is shown the distribution of cells in accordance with their similarities relative to each variables values. SOM cells gather clubs with similar values and each panel in Fig 5 display the distribution of these variables values across the cells. As a consequence, the relationship between the variables can be explored. It is apparent the approximate correlation between a club’s transfer spending and UEFA points. Moreover, it is interesting to note that the maximum values of relative transfer spending (upper left corner in the *Relat Spend* map) differ from the maximum values of transfer spending and UEFA points (lower left corner in the *Spend* or *UEFA Points* maps) but it is more similar with domestic ranking (upper left corner in the *Domestic Rank* map). It should be noted that similar domestic rank values are obtained for different clubs in different domestic leagues (there are obviously 21 winner clubs, 21 in second place, etc. which explains the large number of cells with maximum values (red colors).

**Fig 6:**
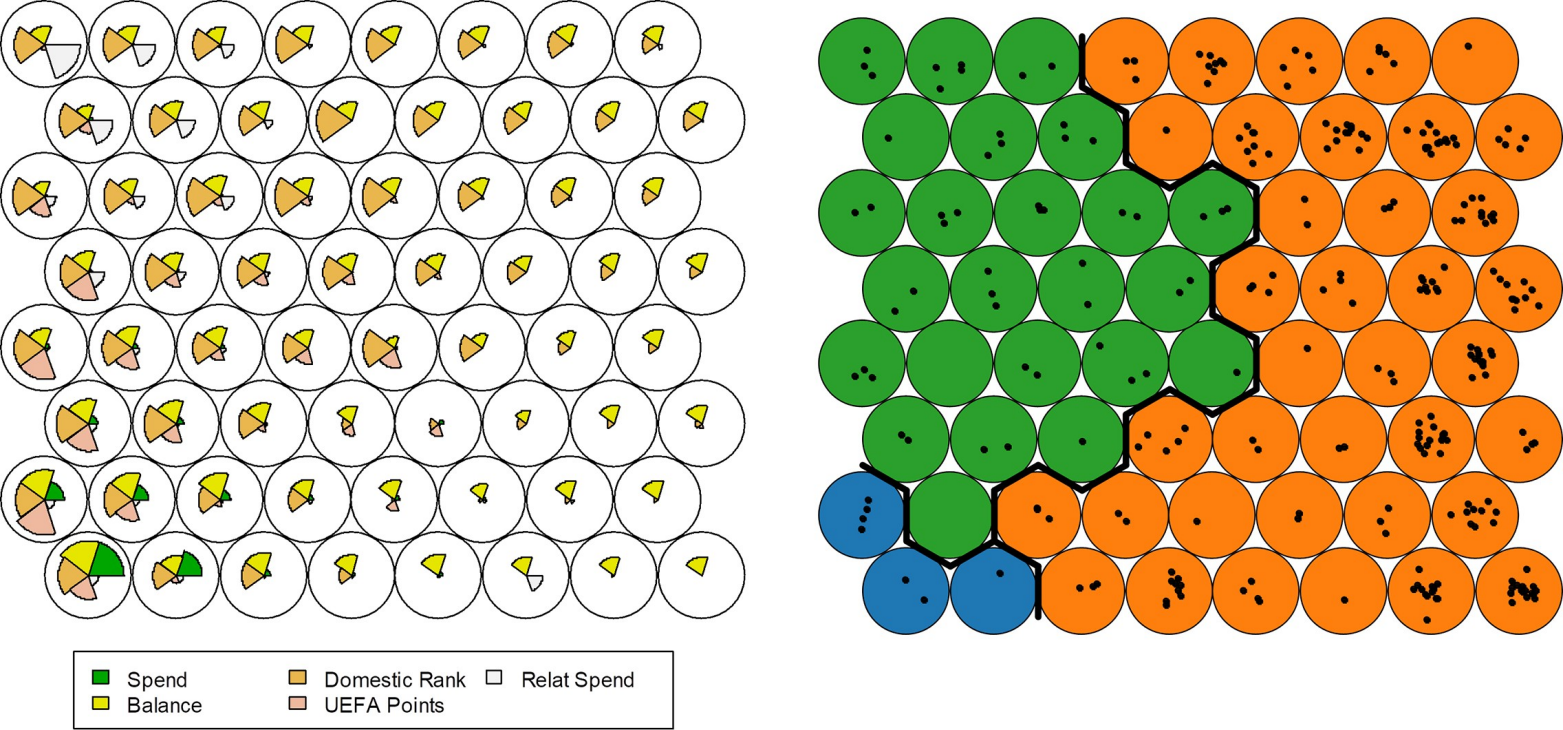
SOM map of codes and clusters for season 2001/2002. The left panel shows the SOM map of codes indicating the underlying distribution of variables involved. The size of each piece of the chart represents the importance of the related variable. The right panel shows the number of clubs belonging to each cell and the optimal number of clusters. This right panel preserves the topological distribution obtained in the left panel.

A more complete perspective of this season is obtained by plotting the SOM map of codes, as displayed in the left panel of Fig 6. As such, a comprehensive picture of variable distributions along the cells set is created. Thereby, every pie chart inside these cells shows the vector of weights of each variable, and every map cell represents regions with similar characteristics. In this way, clubs with similar characteristics belong to the same or adjacent cells. This map distinguishes the clubs’ characteristics along the entire population, taking into account the variables’ importance and closeness. For instance, SOM cells in the lower left corner of the left panel of Fig 6 show the existence of a high correlation between transfer spending, transfer balance, UEFA points, and domestic rank. This implies that clubs belonging to these cells will also reveal these characteristics. Further, confirming the maps of Fig 5, a high correlation between domestic rank and relative transfer spending exists as displayed in the cells in the upper left corner. For further information, the number of clubs belonging to these cells is shown in the right panel of Fig 6. Every black dot inside the cells represents a club. For instance, only two clubs belong to the cell in the lower left corner where the correlations between the examined variables is the highest. Finally, the characteristics’ distributions served as basis for naturally grouping the clubs by performing a clustering partition. For this, we firstly determined the optimal number of clusters (function NbClust() from the R package NbClust [21]). Secondly, the Ward method was used to perform hierarchical clustering resulting in three–the optimal number–clusters overall.

In Fig 6 (right panel), the obtained clusters are displayed with uniform color codes along the cells delimited with solid lines. Comparing both panels, the blue cluster (with only seven clubs) revealed high correlations between domestic rank, transfer balance, transfer spending, and UEFA points, of which transfer spending seems to be the dominant variable. For this specific year, we identified Real Madrid and FC Barcelona from Spain, AC Venezia, Atalanta Bergamo, and Juventus Turin from Italy, Manchester United from England, and Budapest JH from Hungary in this cluster. In contrast, the green cluster is comparably larger, and both transfer spending and UEFA points are clearly much less important variables for the respective clubs. In fact, for most clubs these two variables do not exist at all while the transfer balance, domestic rank, and relative transfer spending are the most important variables driving the configuration of this cluster. As stated above, for some clubs the relative transfer spending is an influential variable connected to domestic ranking. Finally, the orange cluster is the largest in terms of the number of clubs. In this cluster, we identify many clubs that are not able to achieve UEFA points and only achieve low domestic performance (lower right corner of both panels).

**Fig 7:**
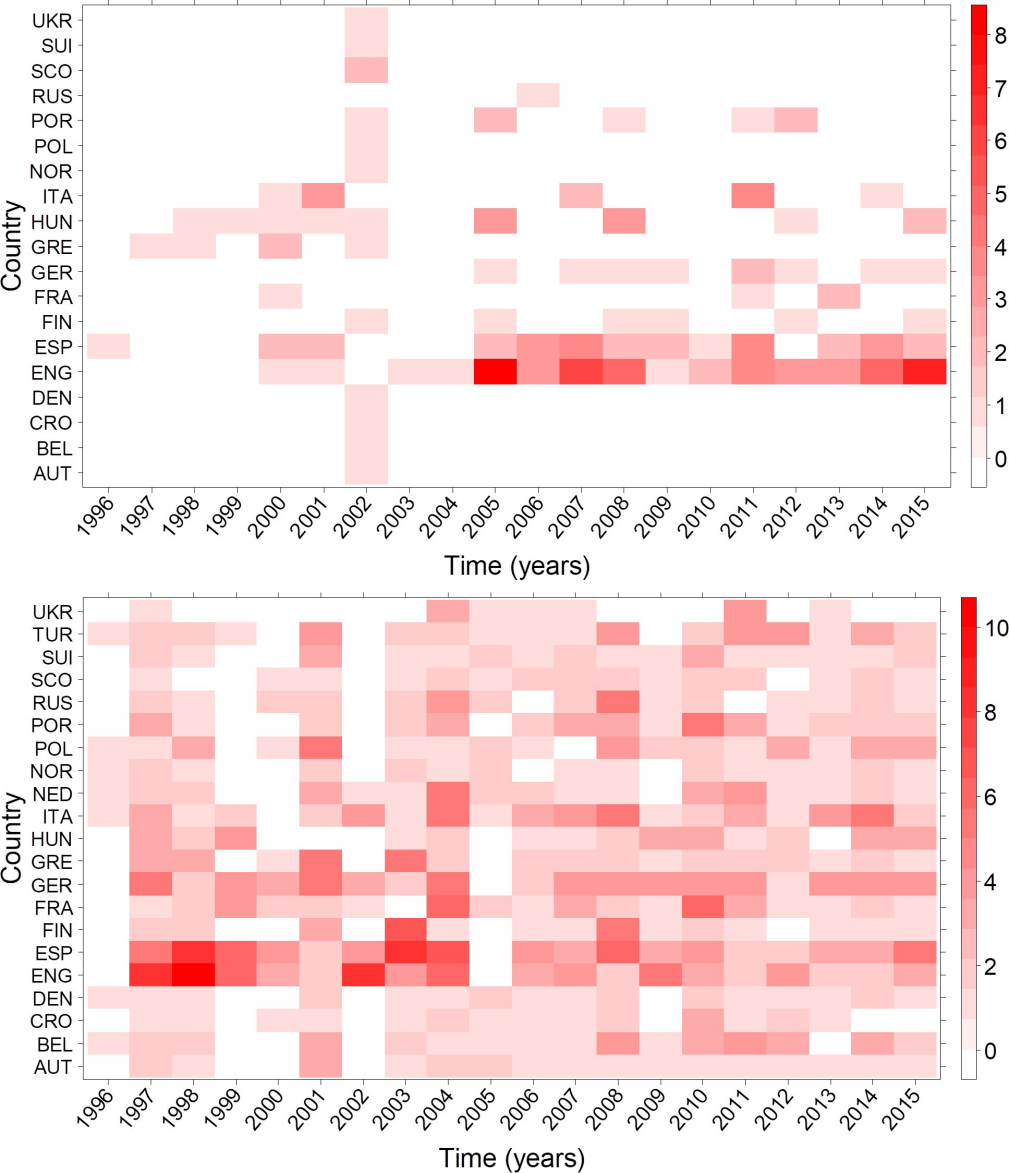
Evolution in the number of clubs in the blue and green cluster. Red squares represent the number of clubs of each league belonging to the blue (upper panel) or green (lower panel) cluster. Years in the x-labels correspond to the first year of the season (i.e. 1996 for 1996–1997 season).

To examine the evolution of these variables, we repeated the calculations for every season. Fig 7 (upper panel) depicts the number of clubs belonging to the “blue” cluster (i.e. with high correlations between domestic rank, transfer balance, transfer spending, and UEFA points) according to their corresponding leagues. This cluster enlarges in the number of clubs and, to a lesser extent, in the number of leagues over time. We further observe that the Premier League contributes the largest number of clubs to this cluster, followed by Spain. The next most well represented first leagues are those from Germany, Portugal, Finland, Italy, France and Hungary. The remaining leagues are only represented by one club—except for Turkey and Netherlands—which surprisingly are not represented at all in the blue cluster. These results suggest that variables driving this cluster have increased in their importance over time, and transfer spending is therefore gaining relevance in achieving European and domestic sportive performance. The blue cluster consists of 142 club observations during the entire time period. However, these observations correspond to only 71 unique clubs, hence, many of them appear more than once in the blue cluster over time. For instance, Real Madrid is found in the blue cluster with 13 observations, followed by Manchester United and FC Chelsea (9 observations each). In fact, only 10 clubs make up 50% of all observations (72 times) in the blue cluster. Ordered by the number of observations, clubs belonging to the blue cluster are: Real Madrid, Manchester United, FC Chelsea, FC Barcelona, Manchester City, FC Liverpool, Atlético Madrid, Bayern Munich, Tottenham Hotspur, Juventus Turin. This cluster is thus formed by the most successful and renowned clubs both at a domestic and European level and, therefore, we name it ‘top cluster’. In contrast, the lower panel in Fig 7 presents the same information for the green cluster. Here, clubs from all leagues are represented with different frequencies.

**Fig 8:**
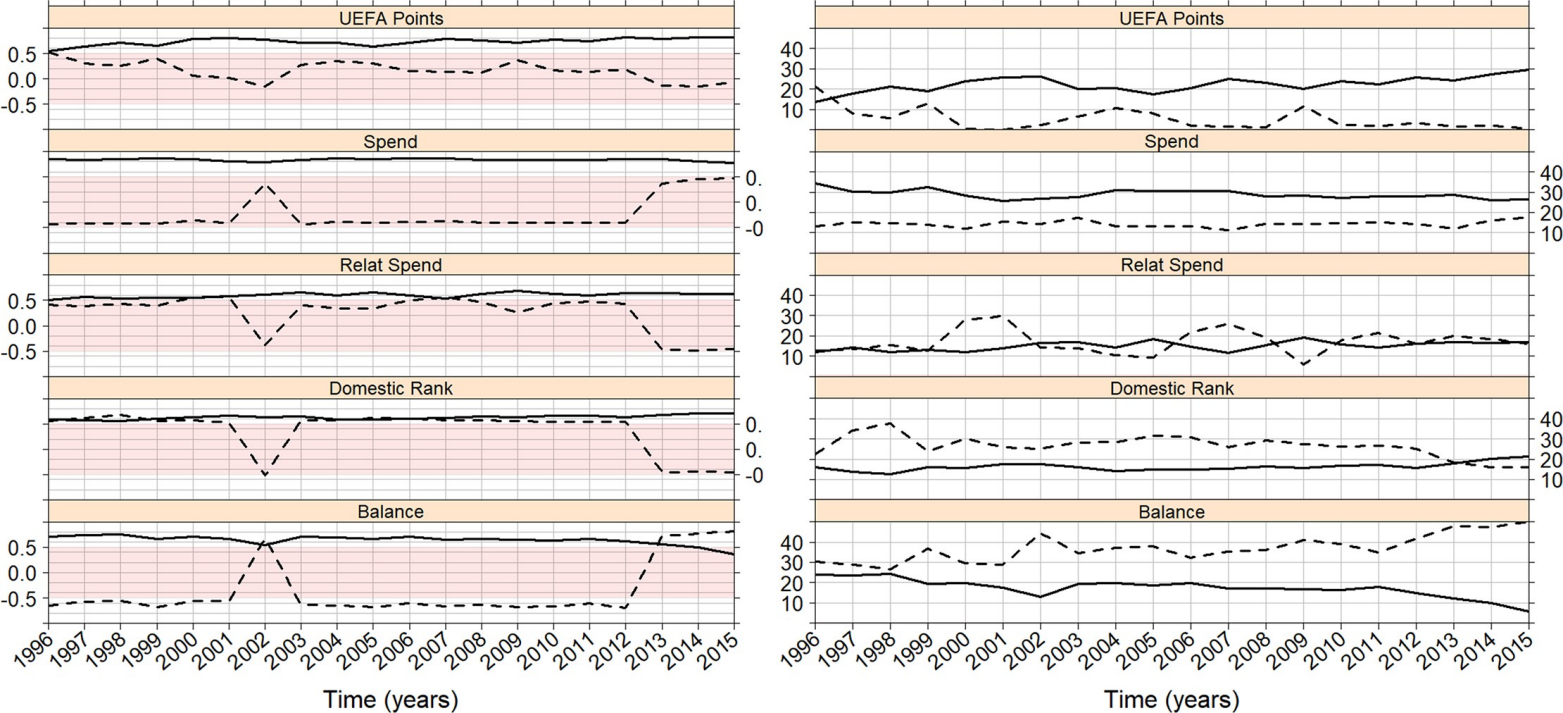
Principal Component Analysis. Correlations of the five variables with the first (solid lines) and second (dashed lines) component displayed in the left panel. The right panel shows the percentage of variance of each variable for the first (solid lines) and the second (dashed lines) components. Years in the x-labels correspond to the first year of the season (i.e. 1996 for 1996–1997 season).

Finally, Fig 8 shows the results derived from the PCA. A principal decomposition was performed in every season, resulting in a corresponding set of eigenvalues. For every season across the entire time period, the first two components retain more than 70% of the data variance with the first component retaining around 50% of the variance. A representation of the variables’ correlations with the first and second components is displayed in the left panel of Fig 8. Light red areas indicate lower (not deemed important) values of correlation (less than 0.50). The correlations of the variables with the first component (solid lines) clearly show transfer spending as the driving variable (0.78 to 0.89), followed by UEFA points (increasing from 0.52 to 0.88). Both domestic rank and relative spending follow in importance with their corresponding correlations increasing along the time period (from 0.50 to 0.75 approximately). In contrast, the transfer balance seems to decrease in importance over time, since its correlation with the first component decreases from 0.71 to 0.32. Correlations with the second component (dashed lines) do not reveal a driving variable as only the transfer balance displays a relatively high correlation (0.80), but this is only at the end of the time period. However, the correlation of all variables with the second component in year 2002 is worth mentioning. It seems to be related with the abrupt drop in transfer spending made by most of the big European leagues, as displayed in S3 Fig.

Although correlation with components is an important variable in every PCA, it is necessary to also consider how much variance is explained by every principal component. This is shown in the right panel of Fig 8. Again transfer spending and UEFA points are both variables that hold a greater percentage of first component variance (solid lines). Moreover, UEFA points increase in importance from 13% to 30%. Similar to the correlation analysis, the transfer balance decreases in importance during the period to 5%. The percentage of variance with respect to the second component (dashed lines) shows that the transfer balance and the domestic rank are the most important variables.

## Concluding remarks

This paper employs a dynamic network approach to analyze the transfer market activities among 21 European first leagues between the seasons 1996/1997 and 2015/2016. We collected transfer records among more than 2,200 clubs, which were involved in more than 135,000 transfers during the time period. In the networks, nodes were clubs and links represented the players transferred from one club to another, which were weighted by the actual fees paid (or received) for each transfer. We extended the work of Liu et al. [12] by analyzing the evolution of the network over time. Additionally, we employed a machine learning approach based on both Kohonen SOMs and PCA to reveal similarities among clubs and cluster them in terms of their transfer market activities. This led to multiple findings from which we derived future research directions.

As a first finding and from a topology perspective, the European transfer network seems to have reached an upper limit in both the number of clubs involved and in the number of players transferred. At approximately the time of the global financial crisis (2007/2008) these numbers stopped growing and the network became more connected and dense. Note that during the time of the Dotcom crisis in the late 1990s and the 9/11 attacks, the network continues to grow even though the clubs’ transfer spending sharply declines thereafter (as shown in S3 Fig). In contrast to this stagnation in network size, only transfers to and from Asia continued to increase, while all other regions decreased their interaction with the European football market. At the end of the examined time period, the transfer network became less diverse in terms of clubs and leagues compared to the mid-2000s. Remarkably, the whole transfer network evolved towards a small-world network which is accentuated over time.

A second finding focuses on the relationship between transfer market activities and sportive performance. As such, transfer spending is identified as a key factor for success in international UEFA competitions. We find intense rivalry among leagues at the European level but only a few rich clubs can achieve top positions. We furthermore show that transfer spending is the main driver of UEFA and domestic sportive performance, while relative transfer spending and transfer balance are less important. When looking at club clusters we find significant heterogeneity among clubs and leagues as also reported by Liu et al. [12]. However, within the ‘top cluster’ the most important European clubs achieve sportive performance very similarly through transfer spending, which originates mainly from England, Spain, Germany and Italy. As such, club managers might have to reconsider their strategic focus and reevaluate the (financial) resource allocation within their clubs to achieve optimal sportive results.

Further findings can conclusively be derived by looking at the inequality of clubs’ transfer activities, specifically at transfer spending differences in acquiring talent through the transfer market. Although the transfer market is a key factor in achieving sportive performance, at least for the most important leagues in Europe, this connection is far from being perfect. As such, uncertainty about the sportive outcome might still exist. For instance, Rottenberg [22] explicitly state that a roughly equal distribution of talent is needed in order to generate uncertainty of outcome. (For recent studies on outcome uncertainty in football see, e.g., [23] and [24–27]). In turn, this might be relevant for keeping fans interested in both individual matches and countries’ leagues. From our findings, however, it also becomes clear that most European first football leagues seem not to comply with this theoretical axiom as their clubs are characterized by decisive differences in transfer spending behavior and, hence, financial resource endowments. In turn, these differences might affect sportive results since they are crucial in acquiring future talent. Similarly, Rottenberg [22] find theoretical evidence for equal opportunities in European professional football but less uncertainty of outcome at the league level as long as national leagues are dominated by a small number of clubs. We find that the connection between transfer spending and sportive performance, especially in UEFA competitions, is extremely strong particularly for clubs from the ‘top cluster’, which might further limit the overall level of uncertainty of outcome. In addition, such a strong connection produces further opportunities and risks. As improved sportive performance from the acquisition of new talents will in turn lead to higher financial income, it might be possible that a virtuous circle is created. As such, financially well-endowed clubs might also be successful in the long-term. However, this interdependence might also lead to a vicious circle with substantial negative implications. Such developments would further increase inequality within the European football market.

From these findings, we suggest three areas for future research. First, an in-depth examination of lower league levels would broaden the scope of our analyses. For example, the second divisions in England and Germany include clubs with a wide scope of different financial resource endowments, providing further opportunities to examine inequalities within the transfer market. Further, the inclusion of football leagues from Asia, North America, and South America to our dynamic network approach would enable an inter-continental comparison with regards to transfer market activities. Second, further variables such as clubs’ financial indicators (e.g., revenues, profitability, cash flow, leverage) should be examined in future studies. Thereby, a more detailed understanding of sportive performance drivers with regards to network specifics could be obtained while better identifying potential sources for future transfer spending. Third, a detailed investigation of football clubs’ transfer spending behavior during times of economic crises would aid in understanding the direct and indirect connection between the football market and worldwide economic shocks.

## Supporting information

S1 Text Extended explanations on network property measures. Measures included degree (connectivity), average path length, density of links and clustering coefficient.
(DOCX)

S1 Fig Evolution of the transfer market activities with EFL21 clubs (‘core nodes’) for different geographical regions (chained base). Each panel describes the evolution of the number of regional clubs involved in transfer market activities with EFL21 clubs.
(TIF)

S2 Fig Evolution of the network measures (chained base). Different descriptive network measures. Density: density of links/transfers, CC: average (over the network nodes/clubs) Clustering Coefficient, Gsize: number of network links/transfers, Gorder: number of network nodes/clubs, Degree-in: average number of incoming links/transfers per club, Degree-out: average number of outgoing links/transfers per club.
(TIF)

S3 Fig Transfer spending by each league along the entire period. Transfer spending produced by each league, taking into account all bought players by all clubs in the corresponding league/season, for every season and for all leagues.
(TIF)
