# Supplementary material for: Transfer market activities and sportive performance in European first football leagues: A dynamic network approach
Source: PLoS One. 2018 Dec 19;13(12):e0209362. doi: 10.1371/journal.pone.0209362 (PMC6300326; doi:10.1371/journal.pone.0209362)
Supplement: S1 Text — Measures included degree (connectivity), average path length, density of links and clustering coefficient. (DOCX) [file pone.0209362.s001.docx]

**S1 Text. Extended explanations on network property measures.**

The *degree* (or *connectivity*) of a node is defined as the number of links, incoming or outgoing, of this node. The *degree-in* is the number of incoming links while the *degree-out* is the number of outgoing links. A node with high degree implies a high synchronization of the node with other nodes. The node degree is an important centrality measure of a network because nodes with high degree play a central role in the network dynamics.

The *average path length* (APL) is calculated as the average number of steps along the shortest paths for all pairs of network nodes. Low APL values imply efficient and fast communication across the network topology. The average path length is calculated by

$${APL}_{G} = {\frac{1}{n(n-1)}}=\sum_{i\neq j} d (v_{i}, v_{j})$$

Where G is the directed graph with a set of nodes V. {\displaystyle d(v_{1},v_{2})}$d(v_{1,}v_{2})$ {\displaystyle v_{1},v_{2}\in V}denotes the shortest distance between $v_{1,}${\displaystyle v_{1}} and {\displaystyle v_{2}}$v_{2}$. Assuming that {\displaystyle d(v_{1},v_{2})=0}$d\left( v_{1,}v_{2} \right)=0$, then $v_{2}${\displaystyle v_{2}} cannot be reached from{\displaystyle v_{1}} $v_{1}$. *n* is the number of nodes.{\displaystyle l_{G}}

The *density of the links* is defined as the ratio of the actual number of links in the network to the number of all possible links between the network nodes. This measure is ill-defined in a network with multiple links i.e. transfers, between two nodes i.e. clubs, because no maximum number of links exists. A simplified version of the network such that multiple links are eliminated can be constructed in order to confidently calculate this measure. A network with many links or a high density of links implies high connectivity among nodes. The density of links for a simple network is calculated by

$$DoL=\frac{\left| E \right|}{\left| V \right|(\left| V \right|-1)}$$

where |*E*| is the number of links and |*V*| is the number of nodes in the network.

The *clustering coefficient* (CC) is a measure of the degree to which nodes in a network tend to group together. Here, we use the definition by [1], which indicates a local vertex-level quantity. The CC is calculated by

$$C_{i}^{w}= \frac{1}{s_{i}(k_{i}-1)}\sum_{j,h} \frac{w_{ij}+w_{ih}}{2}a_{ij}a_{ih}a_{jh}$$

Where $s_{i}$, is the strength of vertex *i*, $a_{ij}$ are elements of the adjacency matrix, $k_{i}$ is the vertex degree, and $w_{ij}$ are the weights. The strength of vertex *i* is measured by the strength of vertices in terms of the total weight of their connections.

Overall, the CC is a measure of local cohesiveness that takes into account the importance of the clustered structure on the basis of the amount of interaction intensity actually found on the local triplets (see [1] for a deeper explanation on this measure).

**References**

1. Barrat A, Barthelemy M, Pastor-Satorras R, Vespignani A. The architecture of complex weighted networks. Proceedings of the national academy of sciences. 2004; 101: 3747–3752. doi: 10.1073/pnas.0400087101.
