# Supplementary figures and images for: Transfer market activities and sportive performance in European first football leagues: A dynamic network approach
Source: PLoS One. 2018 Dec 19;13(12):e0209362. doi: 10.1371/journal.pone.0209362 (PMC6300326; doi:10.1371/journal.pone.0209362)

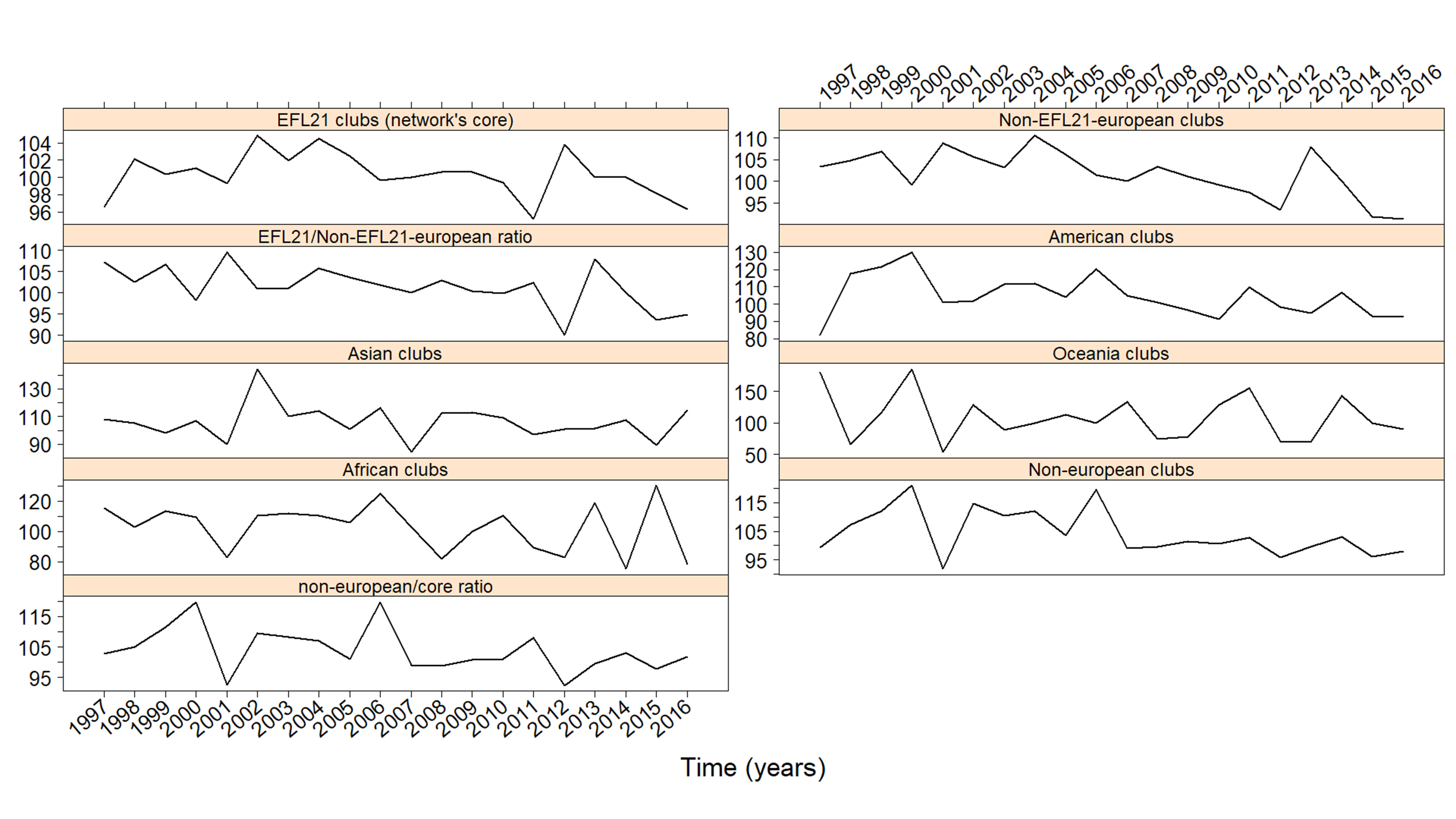

Supplement: S1 Fig — Each panel describes the evolution of the number of regional clubs involved in transfer market activities with EFL21 clubs. (TIF) [file pone.0209362.s002.tif]

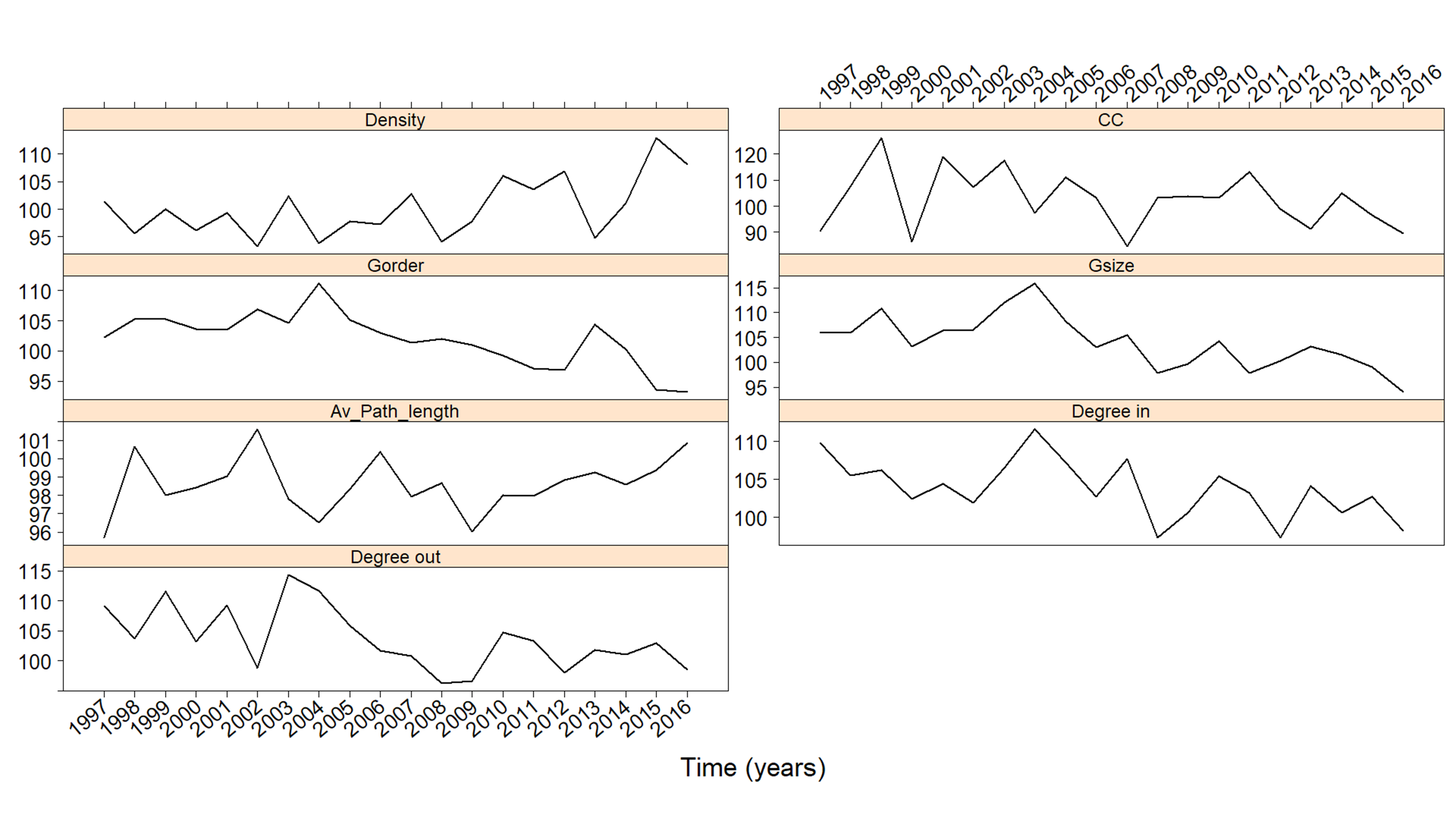

Supplement: S2 Fig — Different descriptive network measures. Density: density of links/transfers, CC: average (over the network nodes/clubs) Clustering Coefficient, Gsize: number of network links/transfers, Gorder: number of network nodes/clubs, Degree-in: average number of incoming links/transfers per club, Degree-out: average number of outgoing links/transfers per club. (TIF) [file pone.0209362.s003.tif]

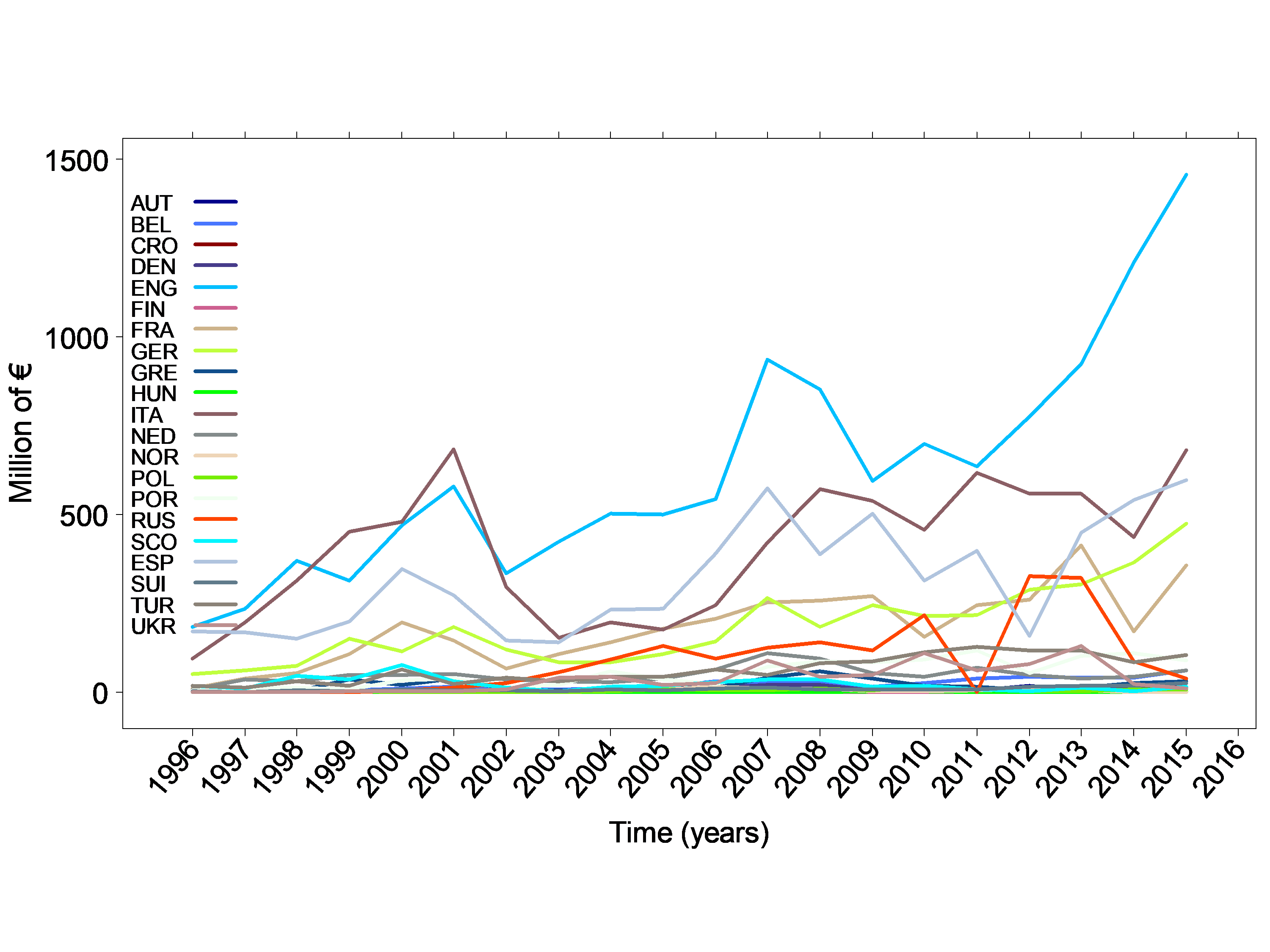

Supplement: S3 Fig — Transfer spending produced by each league, taking into account all bought players by all clubs in the corresponding league/season, for every season and for all leagues. (TIF) [file pone.0209362.s004.tif]
